# Supplementary material for: Genome-wide association and multi-omic analyses reveal ACTN2 as a gene linked to heart failure
Source: Nat Commun. 2020 Feb 28;11:1122. doi: 10.1038/s41467-020-14843-7 (PMC7048760; doi:10.1038/s41467-020-14843-7)
Supplement: Supplementary file 1 — Supplementary Information [file 41467_2020_14843_MOESM1_ESM.pdf]

Genome-wide association and multi-omic analyses reveal *ACTN2* as a gene linked to Heart Failure

Arvanitis et al.

Supplementary Information

Supplementary Figure 1

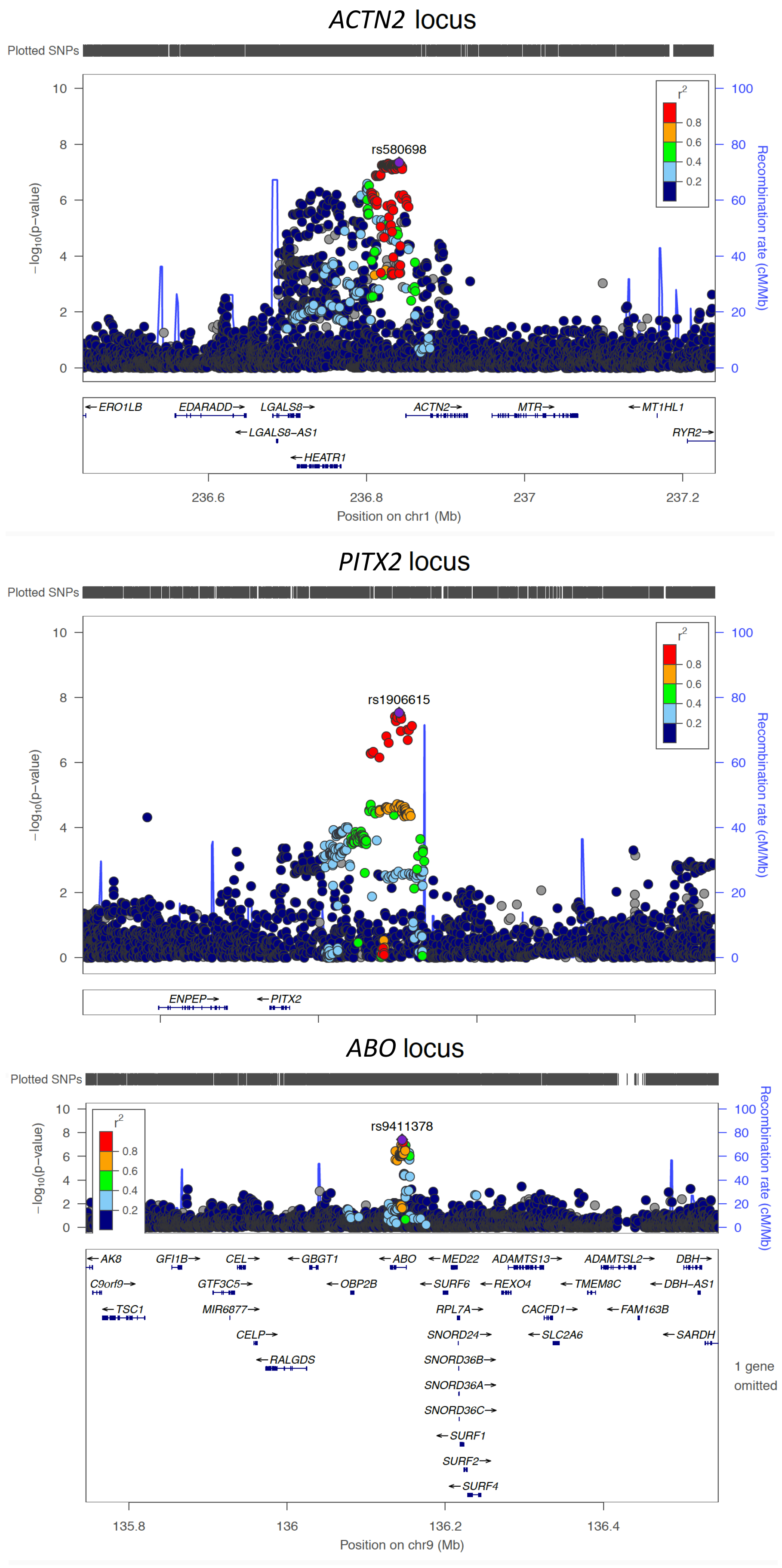

Supplementary Figure 1. LocusZoom plots for the genome-wide significant loci. SNPs are colored based on their LD with the sentinel locus SNP on the 1000 Genomes European reference panel. Chromosomal positions are based on hg38. N=448,549 independent participants.

Supplementary Figure 2

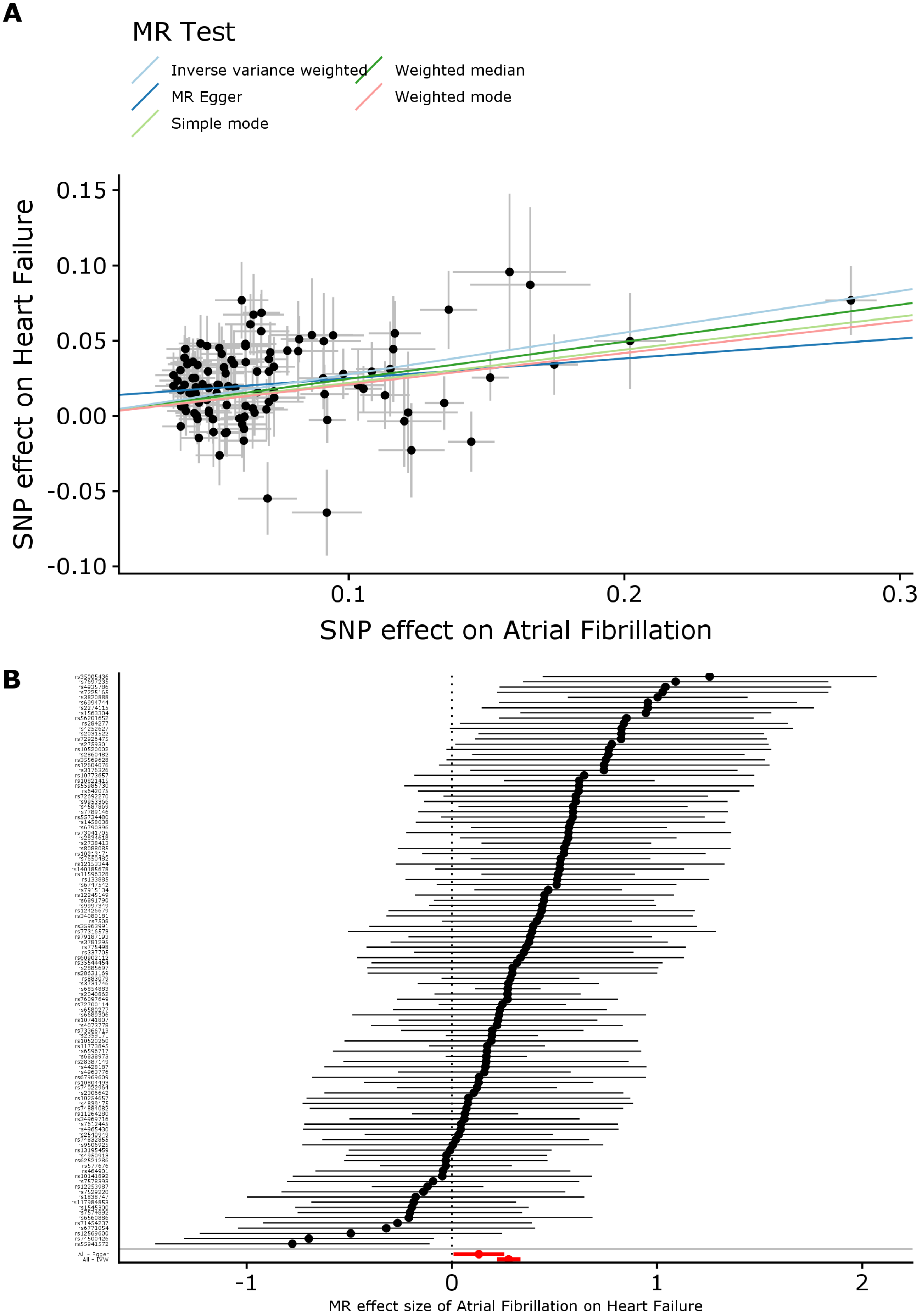

Supplementary Figure 2. Mendelian Randomization of the effect of Atrial Fibrillation on Heart Failure. **A.** Mendelian Randomization plot for the effect of genome-wide significant Atrial Fibrillation SNPs on Heart Failure. The effect size (logOR) of each SNP on Atrial Fibrillation is on the x axis, whereas the y axis shows the effect size of each SNP on Heart Failure. **B.** Mendelian Randomization Forrest plot for all individual instruments analyzed. Error bars represent the 95% confidence intervals of the estimate. Source data are provided as a Source Data file.

Supplementary Figure 3

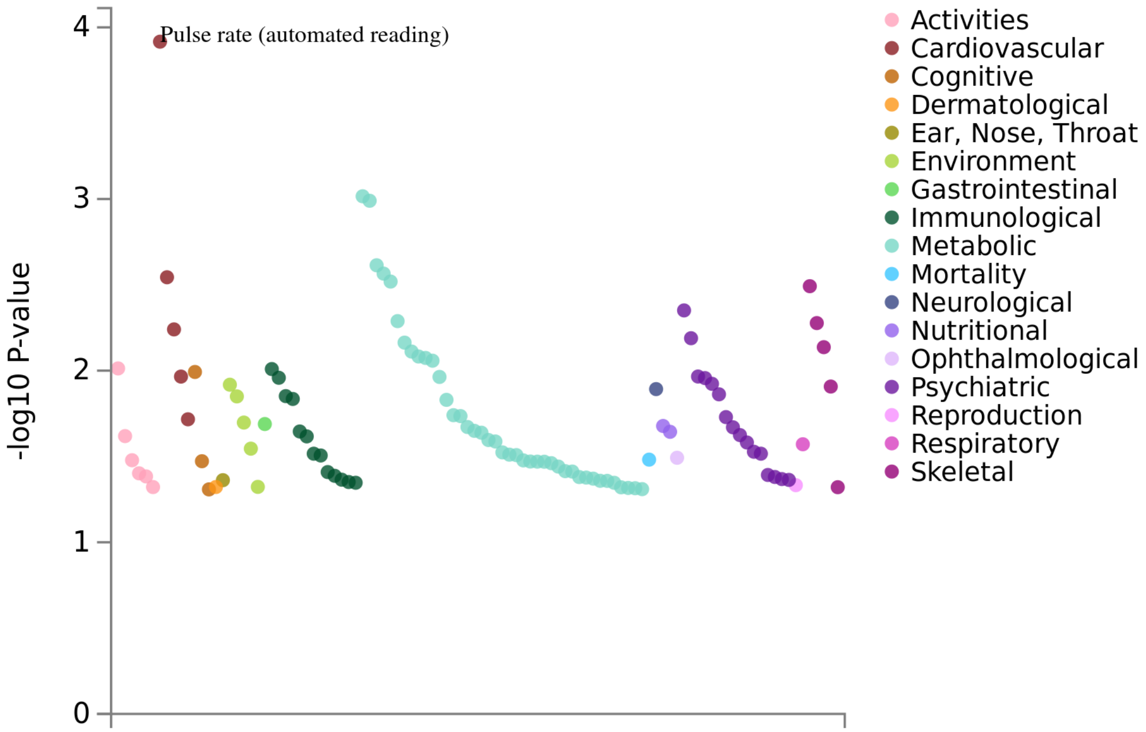

Supplementary Figure 3. PheWAS of the fine-mapped *ACTN2* locus variant (rs535411) in the GWAS Atlas. No association is significant after Bonferroni correction.

Supplementary Figure 4

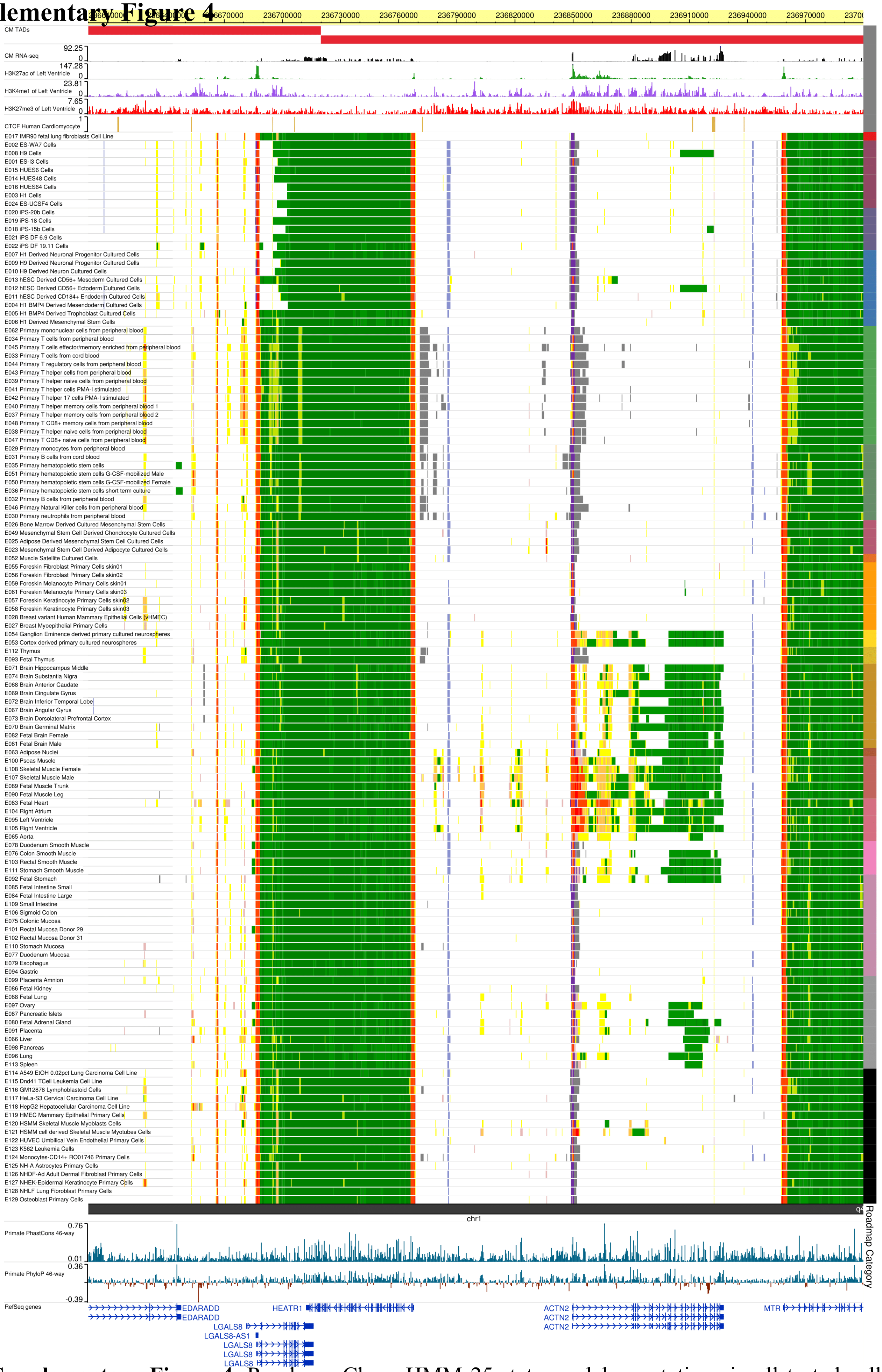

Supplementary Figure 4. Roadmap ChromHMM 25-state model annotations in all tested cell-types and tissues, H3K4me1, H3K4me3, H3K27ac peaks in left ventricle, cardiomyocyte topologically associated domains and PhyloP and PhastCons evolutionary conservation values.

Supplementary Figure 5

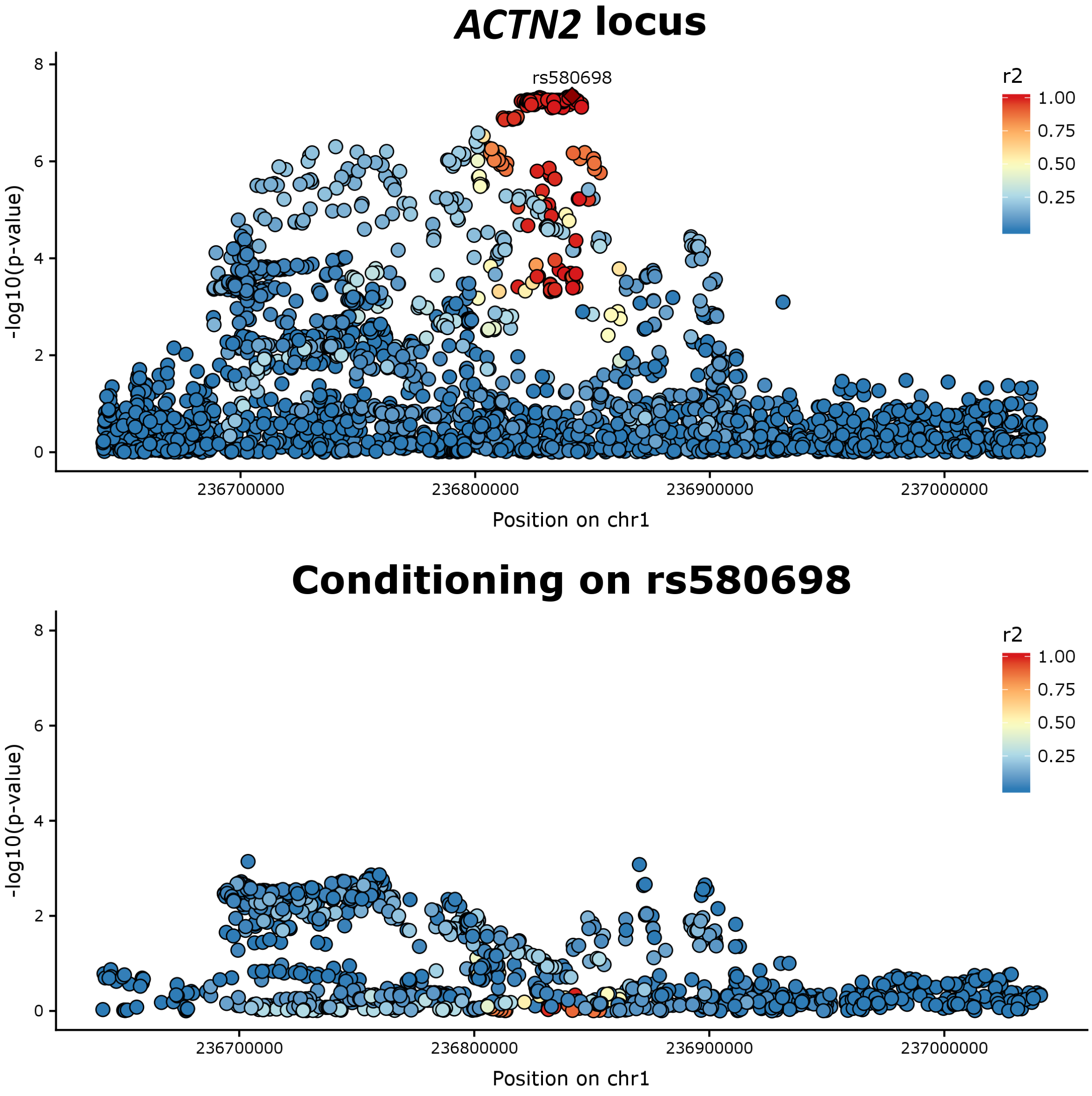

Supplementary Figure 5. Conditional analysis in the *ACTN2* locus. *ACTN2* locus Manhattan plot before (upper panel) and after (lower panel) conditioning on the sentinel locus variant (rs580698).

Supplementary Figure 6

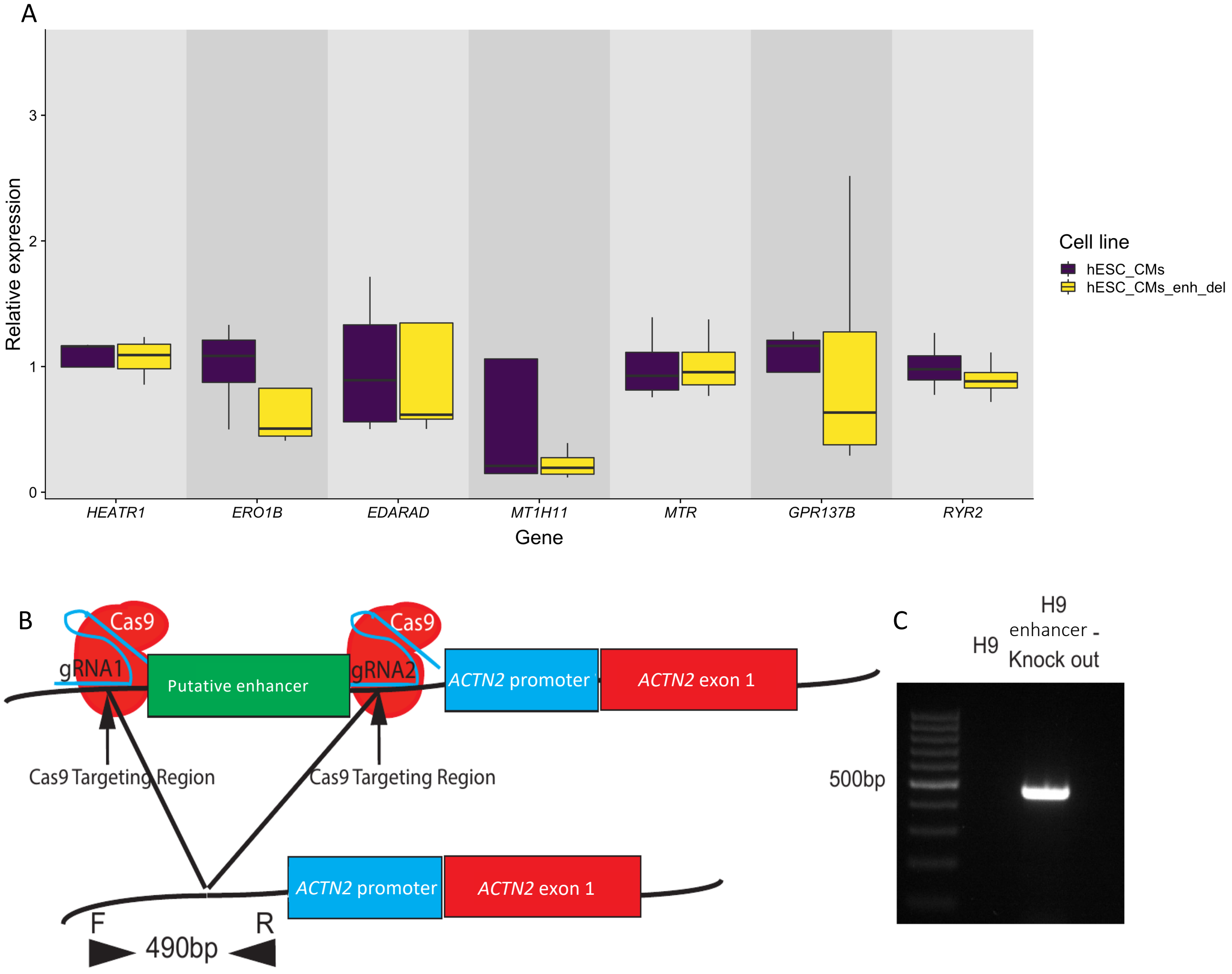

Supplementary Figure 6. Genome editing of the putative *ACTN2* locus enhancer. **A.** Differential expression of genes within 1Mb of the *ACTN2* locus sentinel SNP on Day 15 of cardiomyocyte differentiation between genome edited myocytes that carry the enhancer deletion and isogenic controls. All comparison p-values are >0.4 (n=4 independent biological replicates). Boxplot center line represents the median, the bounds represent the interquartile range (IQR) (25%-75%) and the whiskers extend from the bounds to the largest value no further than 1.5*IQR from the bound. Data beyond the end of the whiskers are plotted individually. **B.** Schematic description of the CRISPR-Cas9 experiments. **C.** PCR of the edited cell lines show a ~500bp fragment consistent with the deleted enhancer region. Source data are provided as a Source Data file.

Supplementary Figure 7

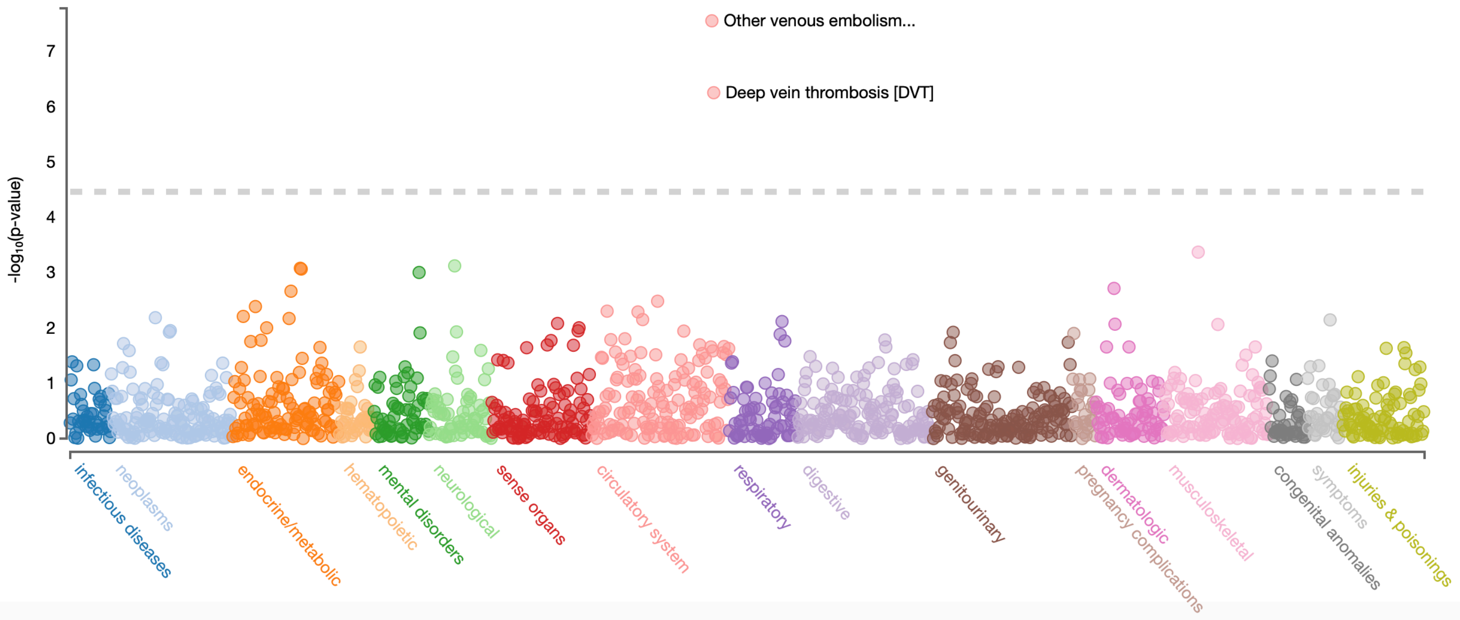

Supplementary Figure 7. PheWAS of the *ABO* locus sentinel variant (rs9411378) across 1,448 traits from the UK Biobank.

Supplementary Figure 8

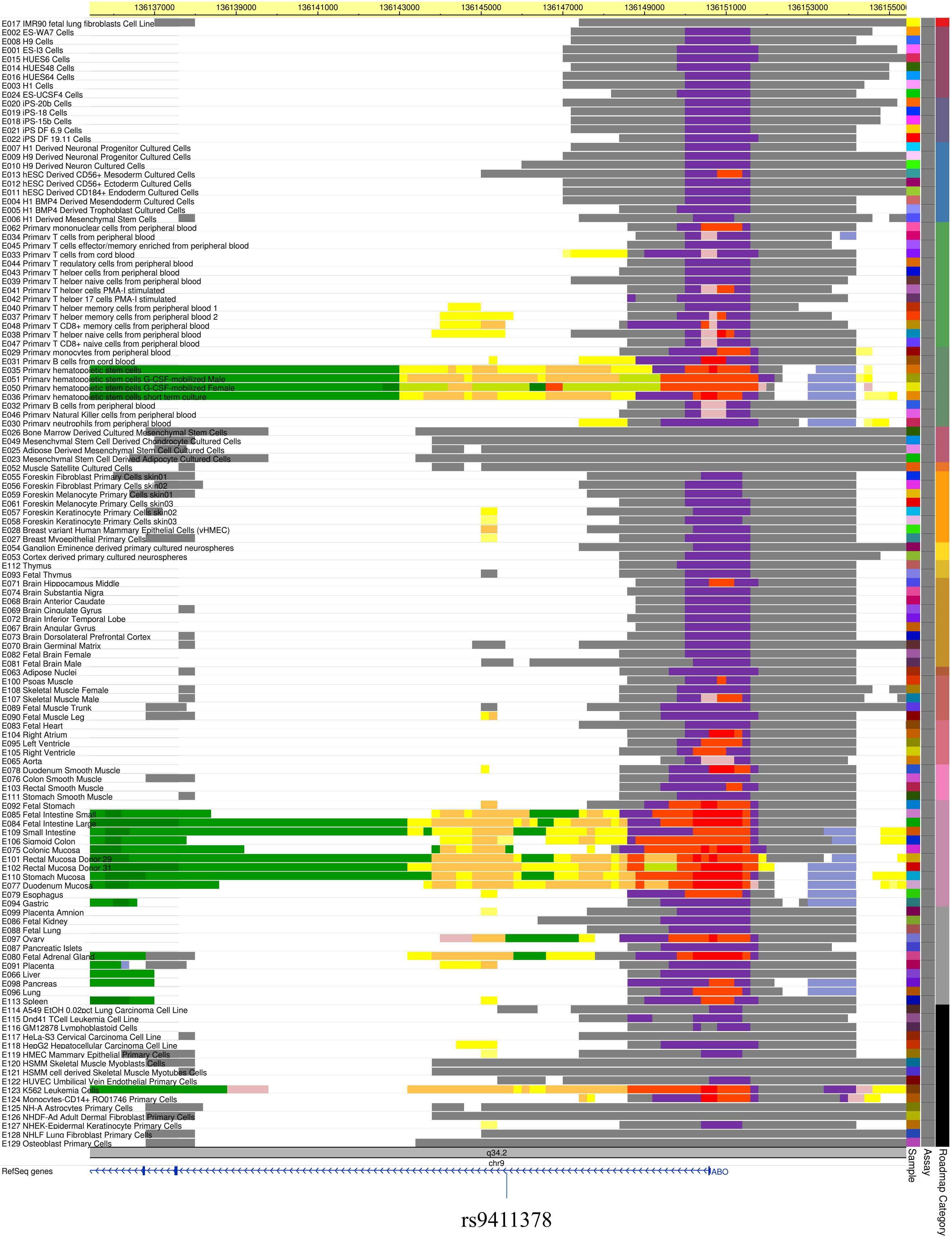

Supplementary Figure 8. Roadmap Epigenomics ChromHMM 25-state model across all tested tissues and cell types for the *ACTN2* locus. Visualization spans ±10KB from the sentinel locus variant.

Supplementary Figure 9

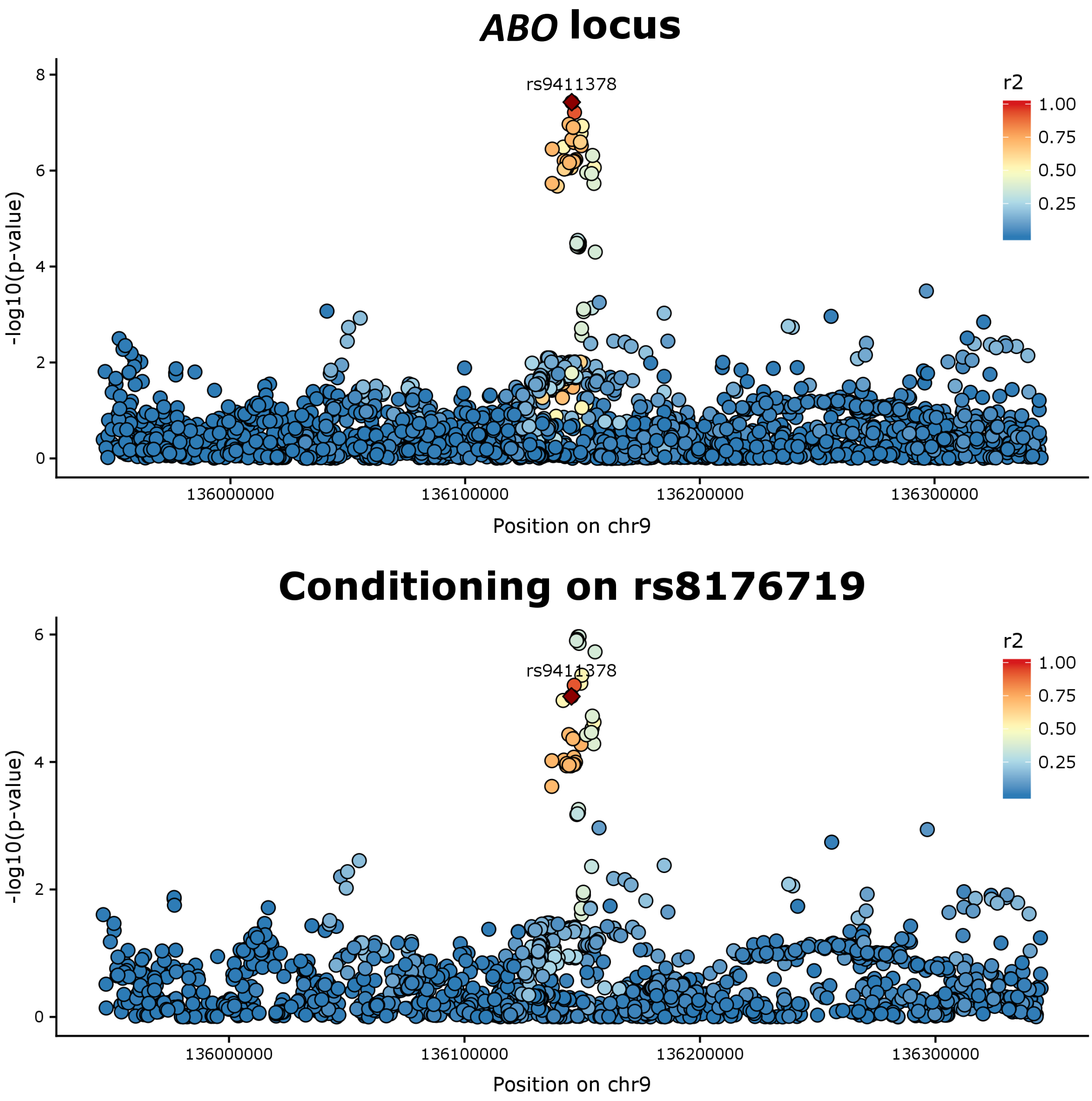

Supplementary Figure 9. Conditional analysis of the *ABO* locus. *ABO* locus Manhattan plot before (upper panel) and after (lower panel) conditioning on the frameshift variant (rs8176719) that determines blood type O.

Supplementary Figure 10

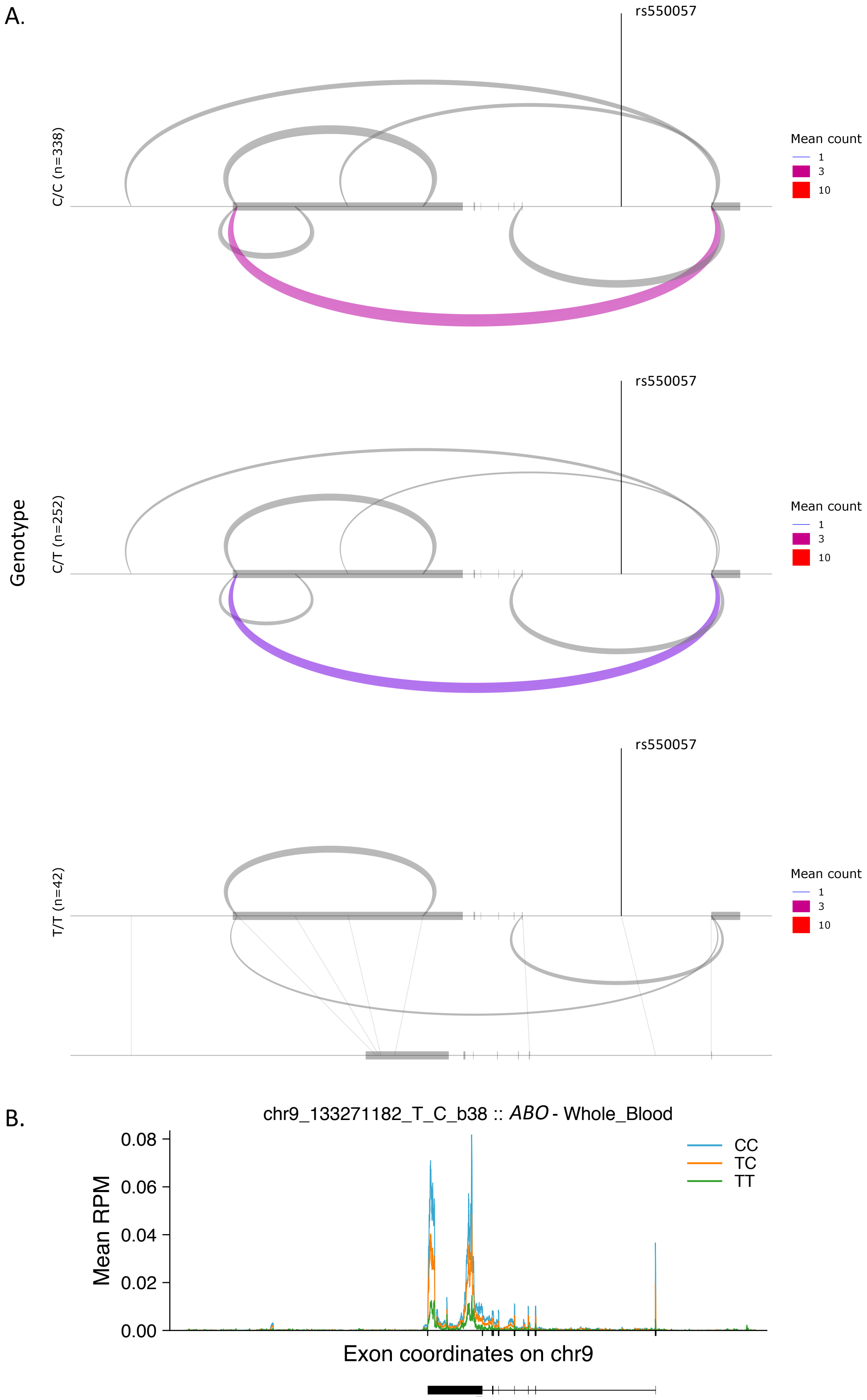

Supplementary Figure 10. Chromosome 9 locus variants affect splicing of the *ABO* gene. **A.** Differential splicing plot in GTEx v8 Whole Blood stratified based on the genotype of rs550057 (variant tagging our sentinel variant rs9411378 (LD r^2=0.92 in 1000 Genomes Europeans)) . We see that the C allele leads to increased expression of all splice variants of the ABO gene overall but disproportionate increase in the expression of the splicing variant denoted by the lowest arc. **B.** Read pile-up plot of the ABO gene in Whole Blood stratified by the genotype of rs550057. Source data are provided as a Source Data file.

Supplementary Table 1. Demographic comparison between the discovery and replication cohorts

	Discovery cohort	Replication cohort
**Sex**	57% female	54% female
**Age <30 years**	1%	14%
**Age 30-45 years**	1%	26%
**Age 45-60 years**	24%	27%
**Age >60 years**	73%	33%
**Non-US based**	91%	<5%

*Although it is unlikely based on the relative demographics of cohort participants, we should note that we cannot exclude the possibility that a small percentage of our Discovery cohort participants are also part of the 23andMe cohort

Supplementary Table 2. Genetic correlation results

Trait 1	Trait 2	Category	rg	se	z	P-value	h2_obs	h2_obs_se	h2_int	h2_int_se	gcov_int	gcov_int_se
CHF	CHF	Cardiovascular	1	2.64E-07	3783000	0	0.0091	0.0011	0.9901	0.0057	0.9901	0.0057
CHF	Cardiac.Dysrythmias	Cardiovascular	0.5219	0.06598	7.91	2.58E-15	0.018	0.002	1.023	0.0089	0.2002	0.0051
CHF	Hypertension	Cardiovascular	0.6791	0.0529	12.84	1.01E-37	0.0737	0.0038	1.1051	0.0141	0.1145	0.0065
CHF	Ischemic.Heart.Disease	Cardiovascular	0.6312	0.05276	11.96	5.54E-33	0.034	0.0025	1.0388	0.0088	0.209	0.0053
CHF	Diseases.of.Connective.Tissue	Dermatologic	0.2329	0.1098	2.12	0.03401	0.0062	0.0012	1.039	0.006	0.0083	0.0044
CHF	Hair.Diseases	Dermatologic	0.3448	0.1343	2.568	0.01021	0.0046	0.0019	1.094	0.0068	0.0026	0.0044
CHF	Sebaceous.Gland.Diseases	Dermatologic	0.3048	0.1056	2.885	0.003916	0.0067	0.002	1.098	0.0068	5.00E-04	0.0044
CHF	Diabetes.Mellitus	Endocrine	0.5307	0.05608	9.465	2.95E-21	0.0361	0.0023	1.0492	0.0097	0.0884	0.0054
CHF	Hypothyroidism	Endocrine	0.2053	0.06274	3.272	0.001067	0.0292	0.003	1.0186	0.0097	0.0369	0.0051
CHF	Lipid.Disorders	Endocrine	0.5724	0.05718	10.01	1.38E-23	0.031	0.0028	1.0533	0.0173	0.1245	0.0053
CHF	Abdominal.Hernia	GI	0.3142	0.06545	4.8	1.59E-06	0.0159	0.0016	0.8305	0.0061	0.0212	0.0039
CHF	Esophageal.Diseases	GI	0.3024	0.06269	4.824	1.41E-06	0.0239	0.0016	1.0312	0.0075	0.0326	0.0045
CHF	Gastritis.Duodenitis	GI	0.3767	0.08223	4.581	4.62E-06	0.0156	0.0014	1.0203	0.0064	0.0295	0.0049
CHF	Genital.Polyp	GU	0.2997	0.1559	1.923	0.05449	0.0059	0.0022	1.0005	0.0061	-0.0051	0.0045
CHF	Genital.Prolapse	GU	0.2632	0.07599	3.464	0.0005332	0.0251	0.003	1.0244	0.0074	-7.00E-04	0.0047
CHF	Menopausal.Disorders	GU	0.6555	0.3283	1.997	0.04585	0.0029	0.002	1.025	0.0058	-0.0063	0.0045
CHF	Iron.Deficiency.Anemia	Hematologic	0.3937	0.1366	2.882	0.003953	0.0046	0.0012	1.043	0.0062	0.0452	0.0047
CHF	Megaloblastic.Anemia	Hematologic	0.442	0.2389	1.8496	0.0644	0.0018	0.0012	1.082	0.0065	0.0182	0.0044
CHF	Coagulopathy	Hematologic	0.7506	0.6318	1.1881	2.35E-01	0.0011	0.0013	1.151	0.0073	0.0176	0.0046
CHF	Cellulitis/Abscess	Infectious.Diseases	0.4278	0.0969	4.4164	1.00E-05	0.0087	0.0019	0.9963	0.0084	0.0572	0.005
CHF	Pneumonia	Infectious.Diseases	0.6629	0.1629	4.07	4.69E-05	0.0036	0.0012	1.099	0.0061	0.1113	0.0045
CHF	Urinary.Tract.Infection	Infectious.Diseases	0.2907	0.1116	2.605	0.009198	0.004	0.001	0.9097	0.0056	0.0531	0.0037
CHF	Enthesopathies	Musculoskeletal	0.3706	0.08571	4.324	1.54E-05	0.0149	0.0015	1.002	0.0065	0.0082	0.0046
CHF	Intervertebral.Disk.Disorders	Musculoskeletal	0.6221	0.09212	6.754	1.44E-11	0.0098	0.0012	1.152	0.0064	0.0077	0.0044
CHF	Osteoarthritis	Musculoskeletal	0.327	0.06626	4.935	8.01E-07	0.0242	0.0027	1.023	0.0104	0.0376	0.0051
CHF	Benign.Colon.Neoplasms	Neoplasms	0.1318	0.08076	1.632	0.1027	0.0131	0.0015	1.0384	0.0078	0.0165	0.0047
CHF	Breast.Cancer	Neoplasms	0.113	0.07493	1.508	0.1315	0.0137	0.002	1.0237	0.0084	0.0022	0.0043

CHF	Skin.Cancer	Neoplasms	0.1035	0.0667	1.551	0.1209	0.0185	0.0034	1.0159	0.0126	-0.0039	0.0046
CHF	Inflammatory.and.Toxic.Neuropathies	Neurologic	0.3778	0.1449	2.608	0.009116	0.0031	0.0011	0.9734	0.0058	0.0166	0.0042
CHF	Facial.Nerve.Disorders	Neurologic	0.3394	0.2529	1.3423	1.80E-01	0.0013	0.0011	0.9944	0.0055	0.0026	0.0044
CHF	Parkinson's.Disease	Neurologic	0.076	0.1859	0.4089	0.68263	0.0023	0.0012	1.0039	0.0058	0.0071	0.0044
CHF	Alcohol-related.Disorders	Psychiatric	0.25	0.08776	2.848	0.004397	0.0103	0.0013	1.0158	0.0063	0.0476	0.0047
CHF	Mood.Disorders	Psychiatric	0.4146	0.07753	5.348	8.89E-08	0.0168	0.0015	0.9966	0.0066	0.0349	0.0045
CHF	Tobacco.Use.Disorder	Psychiatric	0.4977	0.06799	7.319	2.49E-13	0.0285	0.0018	1.0165	0.0071	0.071	0.0051
CHF	Asthma	Respiratory	0.4767	0.05921	8.05	8.27E-16	0.034	0.0029	1.0352	0.0104	0.0347	0.0048
CHF	Chronic.Airway.Obstruction	Respiratory	0.561	0.07316	7.668	1.75E-14	0.0191	0.0018	1.0021	0.0076	0.0926	0.0045
CHF	Nasal.Septum.Deviation	Respiratory	0.1269	0.1777	0.7142	0.47512	0.0022	0.0012	1.0124	0.0062	0.0061	0.0045
CHF	Cataract	Sensory	0.2831	0.07926	3.572	0.0003548	0.015	0.0015	1.0165	0.0071	0.0275	0.0046
CHF	Glaucoma	Sensory	0.1449	0.09512	1.523	0.1276	0.0086	0.0014	1.0059	0.0069	0.0048	0.0042
CHF	Retinal.Detachment	Sensory	-0.06076	0.09267	-0.6557	0.512	0.0092	0.0014	0.9994	0.0067	0.0039	0.0045
CHF	Atrial.fibrillation.or.flutter	Cardiovascular	0.4949	0.05638	8.778	1.66E-18	0.0261	0.0032	1.0171	0.0112	0.2056	0.0053
CHF	Conduction.disease	Cardiovascular	0.5404	0.1178	4.587	4.49E-06	0.0046	0.0012	1.0128	0.0069	0.1941	0.0046
CHF	Congenital.heart.disease	Cardiovascular	0.2712	0.1857	1.46	0.1442	0.0024	0.0011	0.999	0.0062	0.0845	0.0044

Supplementary Table 3. Colocalization analysis for the *ACTN2* locus. All genes within 1MB from the sentinel locus SNP that passed the standard GTEx filters in at least one tissue were tested.

		**Posterior Colocalization probability**		
**Gene**	**Gene Symbol**	**Whole Blood**	**Heart Left Ventricle**	**Heart Atrial Appendage**
ENSG00000198626	*RYR2*	NA	0.256	5.00E-06
ENSG00000244020	*MT1HL1*	NA	0.0482	0.043
ENSG00000237991	*RPL35P1*	3.00E-02	0.0401	0.0645
ENSG00000116984	*MTR*	1.40E-02	3.57E-02	2.14E-03
ENSG00000230325	*AL359921.1*	6.09E-03	0.0301	2.85E-03
ENSG00000273058	*AL359921.2*	0.028	0.0446	0.0757
ENSG00000223776	*LGALS8-AS1*	2.99E-02	0.0574	2.12E-02
ENSG00000186197	*EDARADD*	0.0327	4.33E-06	0.0951
ENSG00000086619	*ERO1B*	4.43E-05	3.29E-06	3.20E-06
ENSG00000235371	*AL122018.1*	NA	5.62E-02	NA
ENSG00000077585	*GPR137B*	4.33E-06	1.08E-04	4.64E-06
ENSG00000119285	*HEATR1*	1.62E-01	2.54E-02	1.77E-01
ENSG00000077522	*ACTN2*	0.0256	0.0416	1.19E-02
ENSG00000116977	*LGALS8*	3.18E-04	3.11E-04	3.21E-04

Supplementary Table 4. HiC interaction data of the ATAC-seq peak containing rs535411 with nearby gene promoters (within 1Mb of the peak).

**Ensembl_ID**	**Gene Name**	**Start_site.hg19.**	**bin_start**	**bin_end**	**Expected interaction counts**	**Observed interaction counts**	**p-value**	**Bonferroni adjusted p-value**
ENSG00000077522	*ACTN2*	236849754	236845000	236850000	29.7701971	54.1604195	2.22E-05	0.00035486
ENSG00000077585	*GPR137B*	236305832	236305000	236310000	NA	0	NA	NA
ENSG00000086619	*ERO1B*	236445319	236445000	236450000	4.79375661	2.58241296	0.85686779	1
ENSG00000116977	*LGALS8*	236681300	236680000	236685000	8.5275051	3.21627092	0.97045867	1
ENSG00000116984	*MTR*	236958610	236955000	236960000	8.66605039	11.5206499	0.16584347	1
ENSG00000119285	*HEATR1*	236767804	236765000	236770000	15.880334	22.7408381	0.0546157	0.8738511
ENSG00000186197	*EDARADD*	236511562	236510000	236515000	5.36150723	2.79916596	0.90267639	1
ENSG00000198626	*RYR2*	237205505	237205000	237210000	4.74287934	3.23828477	0.69701257	1
ENSG00000223776	*LGALS8-AS1*	236687808	236685000	236690000	8.66605039	10.8190117	0.25522745	1
ENSG00000230325	*RP11-385F5.4*	236713580	236710000	236715000	9.86522201	8.2221694	0.65180184	1
ENSG00000235371	*AL122018.1*	236273361	236270000	236275000	3.88129471	8.16006517	0.01803509	0.2885615
ENSG00000237991	*RPL35P1*	237144637	237140000	237145000	NA	0	NA	NA
ENSG00000244020	*MT1HL1*	237167718	237165000	237170000	NA	0	NA	NA
ENSG00000273058	*GC01P235916*	236700004	236700000	236705000	9.32864823	5.36500371	0.90285862	1
ENSG00000222650	*RNU2-70P*	236431080	236430000	236435000	4.69205217	2.94112492	0.84689934	1
ENSG00000226498	*RPSAP21*	236982934	236980000	236985000	7.86494309	8.1101315	0.38860776	1
ENSG00000237922	*AL450309.1*	236450260	236450000	236455000	4.8687276	5.75373614	0.36101872	1
ENSG00000244457	*ENO1P1*	236646465	236645000	236650000	7.45443987	5.53392386	0.75354255	1
ENSG00000252396	*RN7SKP195*	237284107	237280000	237285000	4.29116606	3.23810148	0.62125562	1

Observed and expected counts are based on average of two replicates on day 80 of cardiomyocyte differentiation. P-values are based on the upper tail of a Poisson distribution with lambda the corresponding expected count number.

NA: Not able to perform estimation because of zero observed interaction counts for that region.

Supplementary Table 5. Splice-eQTL results for rs550057 in GTEx_v8 Whole Blood.

Intron coordinates in hg38	P-value	Normalized effect size
9:133233609:133275162	2.40E-05	-0.261937
9:133251152:133251353	0.001945	-0.204811
9:133251152:133252478	6.87E-08	-0.366985
9:133251152:133275162	0.252871	0.0927417
9:133251487:133275162	2.35E-08	-0.399456
9:133262168:133275162	3.71E-06	-0.30869

*Effect size and p-values correspond to the relative intron excision ratios as calculated by LeafCutter

Supplementary Table 6. Heart Failure Definitions Among the Different Cohorts

Cohort	Heart Failure Definition
ARIC	Hospitalization with a heart failure diagnosis according to ICD codes in any position or a death certificate with death from heart failure in any position
CHS	The participant must have both a congestive heart failure diagnosis by a physician and be under treatment with medications for congestive heart failure
Framingham	A definite diagnosis of congestive heart failure requires that a minimum of two major or one major and two minor criteria* be present concurrently. The presence of other conditions capable of producing the symptoms and signs are considered in evaluating the findings.
MESA	Heart failure presence adjudicated by MESA investigators based on presence of symptoms and imaging findings attributable to heart failure along with a diagnosis of heart failure by a physician and medical treatment for heart failure.
WHI	The participant must have both a congestive heart failure diagnosis by a physician and be under treatment with medications for congestive heart failure
eMERGE	Presence of ICD codes for heart failure and positive mention of heart failure in the participant's problem list based on either natural language processing or a structured problem list.
UK Biobank	Hospitalization with a heart failure diagnosis according to ICD codes in any position

ICD: International Classification of Disease

***Criteria used for heart failure diagnosis in the Framingham Study:**

Major Criteria:

1. Paroxysmal nocturnal dyspnea or orthopnea;
2. Distended neck veins (in other than the supine position);
3. Rales;
4. Increasing heart size by x-ray;
5. Acute pulmonary edema on chest x-ray;
6. Ventricular S(3) gallop;
7. Increased venous pressure > 16 cm H_20;
8. Hepatojugular reflux;
9. Pulmonary edema, visceral congestion, cardiomegaly shown on autopsy;
10. Weight loss on CHF Rx: 10 lbs./5days.

Minor criteria:

1. Bilateral ankle edema;
2. Night cough;
3. Dyspnea on ordinary exertion;
4. Hepatomegaly;
5. Pleural effusion by x-ray;
6. Decrease in vital capacity by one-third from maximum record;
7. Tachycardia (120 beats per minute or more);
8. Pulmonary vascular engorgement on chest x-ray.

Supplementary Table 7. Absolute qPCR normalized expression values and primer list used in the hESC-CM CRISPR experiments

**H9 hESC-CM expression**				
ACTN2	0.028446	0.024499	0.030406	0.019
HEATR1	0.005774	0.005787	0.005843	0.002513
ERO1B	0.000592	0.00052	0.000444	0.000222
EDARAD	0.000125	8.78E-05	3.66E-05	4.22E-05
MT1H11	3.94E-06	7.08E-06	3.81E-06	9.05E-05
MTR	0.005638	0.00621	0.010392	0.007613
GPR137B	0.000488	0.000436	0.000453	0.00015
RYR2	0.009976	0.013559	0.010958	0.008284
**H9 hESC-CMs ACTN2 enhancer deletion expression**				
ACTN2	0.017128	0.017251	0.011103	0.008601
HEATR1	0.004261	0.006151	0.005101	0.005769
ERO1B	0.000204	0.000182	0.000246	0.000733
EDARAD	4.43E-05	3.67E-05	4.56E-05	0.000256
MT1H11	4.01E-06	3.08E-06	6.21E-06	1.03E-05
MTR	0.007663	0.005718	0.006597	0.010266
GPR137B	0.000155	0.000111	0.000329	0.000961
RYR2	0.009262	0.011896	0.007681	0.009613

**PCR Primer List**	
ACTN2 For	ATGGCCTTGGACTCTGTGC
ACTN2 Rev	GGTGTTCACGATGTCTTCAGC
MTR For	CTTGGCCTACCGGATGAACAT
MTR Rev	TGCCACAAACCTCTTAATTCCTG
ERADAD For	CCATTCAAGATACGGAACTCCC
ERADAD Rev	AGCAAGTCACTTATGGTGGGG
ERO1B For	TTCTGGATGATTGCTTGTGTGAT
ERO1B Rev	GGTCGCTTCAGATTAACCTTGT
GPR137B For	CTTGTACTTCACGCAGGTGAT
GPR137B Rev	CCAATTTCCCGTCTTTACCAGC
HEATR1 For	GCCCTCCCTCAAAGTGATGC
HEATR1 Rev	CGCTTCCTTAGGGTCAAATAACA
MT1H11 For	GCAAGTGCAAAAAGTGCAAA
MT1H11 Rev	CCCGGACTTTACGTGTCATT
RYR2 For	ACAACAGAAGCTATGCTTGGC
RYR2 Rev	GAGGAGTGTTCGATGACCACC
rs535411 Screening Primer For	GGGGGTGCTCCTATACCAAT
rs535411 Screening Primer Rev	CTTTCATGGATTGTACTTTTGGTGTTGTATT
