# Supplementary material for: Genome-wide association and multi-omic analyses reveal ACTN2 as a gene linked to heart failure
Source: Nat Commun. 2020 Feb 28;11:1122. doi: 10.1038/s41467-020-14843-7 (PMC7048760; doi:10.1038/s41467-020-14843-7)
Supplement: Supplementary file 3 — Description of Additional Supplementary Information [file 41467_2020_14843_MOESM3_ESM.pdf]

Description of Additional Supplementary Files

File Name: Supplementary Data 1
Description: GWAS variants with p-value < 1e-6 on the Heart Failure GWAS metaanalysis

File Name: Supplementary Data 2
Description: Conditional analysis results

File Name: Supplementary Data 3
Description: Variant fine-mapping for the ACTN2 locus

File Name: Supplementary Data 4
Description: Traits that have a p-value <0.05 for PheWAS association with the finemapped variant rs535411 for the ACTN2 locus

File Name: Supplementary Data 5
Description: Traits that have a p-value <0.05 for PheWAS association with the sentinel variant rs9411378 for the ABO locus
