# Supplementary material for: Genome-wide association and multi-omic analyses reveal ACTN2 as a gene linked to heart failure
Source: Nat Commun. 2020 Feb 28;11:1122. doi: 10.1038/s41467-020-14843-7 (PMC7048760; doi:10.1038/s41467-020-14843-7)
Supplement: Supplementary file 9 — Reporting Summary [file 41467_2020_14843_MOESM9_ESM.pdf]

nature research

Corresponding author(s): Alexis Battle, PhD

Last updated by author(s): 01/17/2019

Reporting Summary

Nature Research wishes to improve the reproducibility of the work that we publish. This form provides structure for consistency and transparency in reporting. For further information on Nature Research policies, see Authors & Referees and the Editorial Policy Checklist .

Statistics

For all statistical analyses, confirm that the following items are present in the figure legend, table legend, main text, or Methods section.

n/a	Confirmed	
☐	☒	The exact sample size (*n*) for each experimental group/condition, given as a discrete number and unit of measurement
☐	☒	A statement on whether measurements were taken from distinct samples or whether the same sample was measured repeatedly
☐	☒	The statistical test(s) used AND whether they are one- or two-sided<br>*Only common tests should be described solely by name; describe more complex techniques in the Methods section.*
☐	☒	A description of all covariates tested
☐	☒	A description of any assumptions or corrections, such as tests of normality and adjustment for multiple comparisons
☐	☒	A full description of the statistical parameters including central tendency (e.g. means) or other basic estimates (e.g. regression coefficient) AND variation (e.g. standard deviation) or associated estimates of uncertainty (e.g. confidence intervals)
☐	☒	For null hypothesis testing, the test statistic (e.g. *F*, *t*, *r*) with confidence intervals, effect sizes, degrees of freedom and *P* value noted<br>*Give P values as exact values whenever suitable.*
☒	☐	For Bayesian analysis, information on the choice of priors and Markov chain Monte Carlo settings
☒	☐	For hierarchical and complex designs, identification of the appropriate level for tests and full reporting of outcomes
☐	☒	Estimates of effect sizes (e.g. Cohen's *d*, Pearson's *r*), indicating how they were calculated

Our web collection on statistics for biologists contains articles on many of the points above.

Software and code

Policy information about availability of computer code

Data collection	Not applicable
Data analysis	Multiple software programs were used in our analyses, including R (v 3.5.1), Python (v 2.7), SNPRelate (v 1.16.0), SAIGE (v 0.35.8), METAL (v 2011-03-25), LD SCore (v 1.0.1), GCTA (v 1.91.7), MatrixEQTL (v 2.2) MRbase (v 0.4.22), coloc (v 3.1), Minimac4 (v 1.0.1), Eagle (v 2.4)

For manuscripts utilizing custom algorithms or software that are central to the research but not yet described in published literature, software must be made available to editors/reviewers. We strongly encourage code deposition in a community repository (e.g. GitHub). See the Nature Research guidelines for submitting code & software for further information.

Data

Policy information about availability of data

All manuscripts must include a data availability statement. This statement should provide the following information, where applicable:

- Accession codes, unique identifiers, or web links for publicly available datasets
- A list of figures that have associated raw data
- A description of any restrictions on data availability

Our GWAS summary statistics were made available in a public Zenodo repository (https://zenodo.org/record/3612522#.XiSE_i2ZOgA), and all the genotype data used to generate those summary statistics are available from dbGaP (accession numbers phs000007.v29.p11 [https://www.ncbi.nlm.nih.gov/projects/gap/cgi-bin/study.cgi?study_id=phs000007.v29.p10], phs000287.v6.p1 [https://www.ncbi.nlm.nih.gov/projects/gap/cgi-bin/study.cgi?study_id=phs000287.v6.p1], phs000209.v13.p3 [https://www.ncbi.nlm.nih.gov/projects/gap/cgi-bin/study.cgi?study_id=phs000209.v13.p3], phs000280.v4.p1 [https://www.ncbi.nlm.nih.gov/projects/gap/cgi-bin/study.cgi?study_id=phs000280.v4.p1], phs000200.v11.p3 [https://www.ncbi.nlm.nih.gov/projects/gap/cgi-bin/study.cgi?study_id=phs000200.v12.p3], phs000888.v1.p1 [https://www.ncbi.nlm.nih.gov/projects/gap/cgi-bin/study.cgi?study_id=phs000888.v1.p1]) or via a request to the UK BioBank. Our analysis of eQTL data from GTEx and eQTLGen are all available in our Supplementary Tables and the corresponding GTEx v8 sequencing data are available from dbGaP (accession number phs000424.v8.p2 [https://www.ncbi.nlm.nih.gov/projects/gap/cgi-bin/study.cgi?study_id=phs000424.v8.p2]) and on the GTEx project portal (https://gtexportal.org/home/). RNA-seq, H3K27ac-seq and Hi-C data from the cardiomyocyte differentiation experiments have been deposited

in the Gene Expression Omnibus under the accession number GSE116862 [https://www.ncbi.nlm.nih.gov/geo/query/acc.cgi?acc=GSE116862] whereas the sequencing raw reads for ATAC-seq and H3K4me1-seq as well as all processed epigenetic, RNA-seq and HiC data in hESC-CMs for our loci of interest were made available at the following Zenodo repository (https://zenodo.org/record/3612522#.XiSE_i2ZOgA). Lastly, the source data underlying Figures 1b, 3a, c, 4c and Supplementary Figures 2, 6a, c, 10a are provided as a Source Data file.

Field-specific reporting

Please select the one below that is the best fit for your research. If you are not sure, read the appropriate sections before making your selection.

☒ Life sciences ☐ Behavioural & social sciences ☐ Ecological, evolutionary & environmental sciences

For a reference copy of the document with all sections, see nature.com/documents/nr-reporting-summary-flat.pdf

Life sciences study design

All studies must disclose on these points even when the disclosure is negative.

Sample size	We performed a large scale GWAS meta-analysis of five cohorts that study cardiovascular disease and two population genetics cohorts, all of European ancestry comprising a total of 10,976 heart failure cases and 437,573 controls
Data exclusions	For each individual study we performed sample level filtering (excluding samples with assigned and genotype sex discrepancy, extreme deviations from heterozygosity or missingness). We also excluded individuals that were not of European Ancestry and for every group of individuals that were related (Identity by descent (IBD) >0.125) we randomly selected one.
Replication	We replicated our findings in an independent cohort of 24,829 Heart failure cases and 1,614,513 controls of European ancestry within the 23andMe research cohort.
Randomization	Not applicable. This is not a randomized trial
Blinding	Not applicable. This is not an intervention trial.

Reporting for specific materials, systems and methods

We require information from authors about some types of materials, experimental systems and methods used in many studies. Here, indicate whether each material, system or method listed is relevant to your study. If you are not sure if a list item applies to your research, read the appropriate section before selecting a response.

Materials & experimental systems

n/a	Involved in the study	
☒	☐	Antibodies
☐	☒	Eukaryotic cell lines
☒	☐	Palaeontology
☒	☐	Animals and other organisms
☐	☒	Human research participants
☒	☐	Clinical data

Methods

n/a	Involved in the study	
☐	☒	ChIP-seq
☒	☐	Flow cytometry
☒	☐	MRI-based neuroimaging

Eukaryotic cell lines

Policy information about cell lines

Cell line source(s)	H9 hESC MLC2v:H2B-GFP reporter transgenic line was generated in Sylvia Evans's lab (Veevers et al. PMID: 30122443) from a wild-type H9 hESC cell line purchased by WiCell.<br>H9 hESC with and without deletion of an identified 1400 bp enhancer region in chromosome 1 were generated in Emmanouil Tampakakis' lab from a wild-type H9 hESC cell line purchased by WiCell.
Authentication	H9 hESC MLC2v:H2B-GFP reporter transgenic line was generated and authenticated in Sylvia Evans's lab by Short Tandem Repeat (STR) profiling analysis.<br>Enhancer-deleted H9 hESC cell lines were authenticated using target PCR experiments in Emmanouil Tampakakis' lab
Mycoplasma contamination	All cell lines used in this study tested negative for Mycoplasma contamination.
Commonly misidentified lines (See ICLAC register)	No commonly misidentified lines were used.

Human research participants

Policy information about studies involving human research participants

Population characteristics	We performed genome wide association studies in five cohorts that study cardiovascular disease (Framingham Heart Study, Cardiovascular Health Study, Atherosclerosis Risk in Communities Study, Multi-Ethnic Study of Atherosclerosis, Women's Health Initiative) and the eMERGE initiative. Genotype and phenotype raw data were downloaded from dbGAP (accession numbers phs000007.v29.p11, phs000287.v6.p1, phs000209.v13.p3, phs000280.v4.p1, phs000200.v11.p3, phs000888.v1.p1)
Recruitment	Each study had its own recruitment characteristics, details of which can be found in the relevant accession numbers provided above.
Ethics oversight	dbGaP data access was approved by our local Johns Hopkins Hospital IRB (IRB00163194)

Note that full information on the approval of the study protocol must also be provided in the manuscript.

ChIP-seq

Data deposition

☒ Confirm that both raw and final processed data have been deposited in a public database such as GEO.

☒ Confirm that you have deposited or provided access to graph files (e.g. BED files) for the called peaks.

Data access links<br>*May remain private before publication.*	Sequencing data from the cardiomyocyte differentiation experiments have been deposited in the Gene Expression Omnibus under the accession number GSE116862
Files in database submission	Too many files to list here. Please check the GEO link.
Genome browser session<br>(e.g. UCSC)	Bed files with RPKM data for the loci evaluated in our GWAS follow-up analyses are provided in a public Zenodo repository (https://zenodo.org/record/3612522#.XiSE_i2ZOgA).

Methodology

Replicates	All ChIP-seq or other profiling experiments were performed on two biological replicates. The biological replicates were two independent differentiation experiments.
Sequencing depth	The ChIP-seq experiments were single-end 50bp. Sequencing depths are 20 million reads at minimum.
Antibodies	Epitope Supplier Cat#<br>H3K27ac Active Motif AM39133<br>H3K4me1 Abcam ab8895
Peak calling parameters	Reads were aligned to hg19 (GRC37) following standards of ENCODE ChIP-seq pipeline. For each stage, ChIP-seq peaks were called using MACS220 for each biological replicate and pooled sample, using the pooled input sample as control. MACS2 was run with the default setting with "--nomodel –extsize 180" parameter. Peaks called in the pooled sample that also intersected with peaks in both replicates by at least 50% bases were defined as replicated peaks and were used as the final peak list for that stage.
Data quality	Ren lab was part of the ENCODE ChIP-seq production center. Sufficient sequencing depth were achieved according to ENCODE standards. Number of peaks for each antibody was compared to published data of corresponding antibody and was quite comparable.
Software	The analysis pipeline is available at: https://github.com/ren-lab/chip-seq-pipeline, and is a simple implementation of ENCODE ChIP-seq pipeline. Software modules used in this pipeline include: BWA, samtools, picard, MACS2, bedtools. Software versions stated earlier.
